# Iron: Regulation, redox homeostasis, and ferroptosis in cancer

**DOI:** 10.70401/fos.2026.0022

**Published:** 2026-03-23

**Authors:** Chesta Jain, Yatrik M. Shah

**Affiliations:** 1 Department of Molecular and Integrative Physiology, University of Michigan, Ann Arbor, MI 48109, USA; 2 Department of Internal Medicine, Division of Gastroenterology and Hepatology, Michigan Medicine at the University of Michigan, Ann Arbor, MI 48109, USA; 3 Rogel Cancer Center, University of Michigan, Ann Arbor, MI 48109, USA

**Keywords:** Iron, oxidative cell death, hypoxia, ferroptosis

## Abstract

Iron is essential for cellular metabolism, redox balance, and proliferation, yet its redox activity generates reactive oxygen species (ROS) that can damage DNA, proteins, and lipids. Cancer cells exploit iron homeostasis mechanisms, including iron regulatory proteins, ferritinophagy, and hypoxia-inducible factors to maintain high intracellular iron, supporting metabolic reprogramming, antioxidant defenses, and therapy resistance. Iron-dependent lipid peroxidation drives ferroptosis, a regulated form of cell death uniquely dependent on iron. Ferroptosis is tightly controlled by metabolic and antioxidant pathways and mitochondrial ROS, as well as by lipid composition and polyunsaturated fatty acid availability. Ferroptosis also intersects with apoptosis and necroptosis, highlighting the central role of iron in cell fate and survival. Dysregulation of these pathways in cancer can sensitize cells to ferroptosis, creating a therapeutic vulnerability. Exploiting ferroptosis through modulation of iron availability, redox defenses, or lipid metabolism offers a promising anticancer strategy. However, tissue-specific iron dynamics, tumor heterogeneity, and interactions within the tumor microenvironment complicate clinical translation. Integrative approaches combining metabolic profiling, genetic analysis, and ferroptosis-targeted interventions will be critical to harness iron-dependent cell death while minimizing systemic toxicity. In this review, we explore the mechanisms through which cancer cells sustain high iron, evading associated toxicities and possible implications for integrating ferroptosis based therapies in clinical oncology.

## 1. Introduction

**Figure 1.**
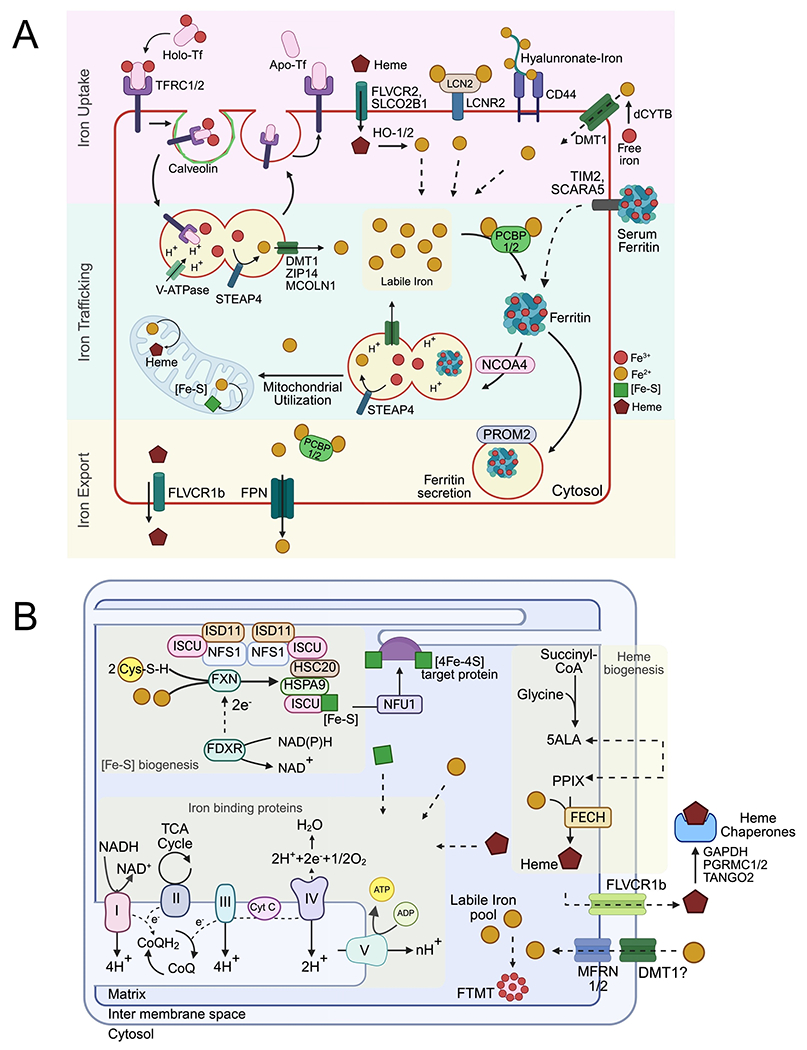
Cellular iron homeostasis. (A) Cellular uptake, trafficking and export; (B) Mitochondrial trafficking and utilization for biogenesis of iron containing cofactors heme and iron sulfur clusters. Created in BioRender.com. CD44: cluster of differentiation 44 (cell surface glycoprotein); DMT1: divalent metal transporter 1 (SLC11A2); FDXR: ferredoxin reductase; FECH: ferrochelatase; FLVCR1b: feline leukemia virus subgroup C receptor 1b; FLVCR2: feline leukemia virus subgroup C receptor 2; FPN: ferroportin (SLC40A1); FTMT: mitochondrial ferritin; FXN: frataxin; GAPD: glyceraldehyde-3-phosphate dehydrogenase; HSC20: heat shock cognate 20; HSPA9: heat shock protein family A (Hsp70) member 9 (Mortalin); ISCU: iron-sulfur cluster assembly enzyme; ISD11: iron-sulfur cluster assembly protein ISD11 (LYRM4); LCN2: lipocalin 2; LCN2R: lipocalin 2 receptor; MCOLN1: mucolipin 1; MFRN1/2: mitoferrin 1/2 (SLC25A37/SLC25A28); NADH: nicotinamide adenine dinucleotide (reduced form); NAD+: nicotinamide adenine dinucleotide (oxidized form); NADPH: nicotinamide adenine dinucleotide phosphate (reduced form); NADP+: nicotinamide adenine dinucleotide phosphate (oxidized form); NCOA4: nuclear receptor coactivator 4; NFS1: NFS1 cysteine desulfurase; NFU1: NFU1 iron-sulfur cluster scaffold; PCBP1/2: poly(rC) binding protein 1/2; PGRMC1/2: progesterone receptor membrane component 1/2; PROM2: prominin 2; SCARA5: scavenger receptor class A member 5; SLCO2B1: solute carrier organic anion transporter family member 2B1; STEAP4: six transmembrane epithelial antigen of prostate 4; TANGO: transport and golgi organization protein (TANGO1, MIA3); TFRC: transferrin receptor (CD71); TIM2: T-cell immunoglobulin and mucin domain-containing protein 2; V-ATPase: vacuolar-type H+-ATPase; ZIP14: zrt- and irt-like protein 14 (SLC39A14).

Iron is an essential micronutrient required for virtually every form of life. Its unique ability to cycle between redox states allows it to participate in diverse biochemical processes, including mitochondrial respiration, DNA synthesis, oxygen transport, lipid metabolism, and regulation of epigenetic and transcriptional programs^[1]^. Despite its critical roles, iron is intrinsically toxic: free ferrous iron (Fe^2+^) catalyzes the generation of reactive oxygen species (ROS), while Fe^2+^ precipitates and becomes biologically inaccessible. To balance these competing demands and evade associated toxicity, organisms have evolved tightly regulated systems governing iron uptake, intracellular distribution, storage, and export at both cellular and systemic levels (Figure 1A).

Highly proliferative cancer cells develop a heightened dependence on iron, a phenomenon often described as “**iron addiction**” since iron not only supports crucial cellular metabolism but is involved in numerous oncogenic signaling pathways^[2]^. Tumor cells exhibit profound metabolic rewiring to sustain proliferation, survive oxidative stress, and to maintain a high intracellular pool even in nutrient-limited microenvironments. These alterations not only enhance cancer cell fitness but also create vulnerabilities that may be exploited therapeutically.

The sections below outline the fundamental mechanisms of cellular iron homeostasis and highlight how cancer cells reprogram these pathways. Together, this framework establishes the biological context for understanding iron-dependent metabolic adaptations and their therapeutic implications.

## 2. Iron Homeostasis

Iron homeostasis is tightly regulated because both deficiency and overload disrupt essential physiological processes. Insufficient iron limits energy production and nucleotide synthesis and slows the regeneration of rapidly renewing tissues such as erythrocytes, gastrointestinal mucosa, airway epithelium, and skin. In contrast, excess iron catalyzes oxidation of proteins, lipids, and DNA, contributing to inflammation and cell death, as seen in hereditary hemochromatosis and other iron overload disorders^[3]^. Systemically, iron homeostasis is achieved through coordinated regulation of dietary iron absorption, systemic distribution, erythropoiesis, and recycling of iron from the senescent red blood cells through reticuloendothelial macrophages. Similarly, at a cellular level, a precise balance among iron uptake, storage, utilization, and export ensures adequate supply while minimizing toxicity. Cancer cells increase iron uptake, restrict export, rewire storage dynamics, and remodel mitochondrial and cytosolic iron utilization to maintain a larger and more reactive labile iron pool (LIP)^[4]^. These adaptations allow tumors to exploit iron-rich microenvironments to sustain proliferation, reprogram metabolism.

### 2.1. Iron uptake

#### 2.1.1 Dietary iron absorption

Iron recycling via degradation of old erythrocytes is very efficient, and normally about 1.1 mg of iron is absorbed from the diet to make up for the obligatory unregulated iron loss due to slouching of intestinal and dermal cells. However, intestinal iron absorption can be increased or decreased during iron deficiency, high erythropoietic demand, or iron overload, respectively^[3]^.

The majority of the iron absorption occurs in the duodenum and proximal jejunum; however, small amounts of iron can be absorbed throughout the small intestine and colon. Luminal ferric iron (Fe^3+^) is first reduced to its ferrous form by the apical ferrireductase duodenal cytochrome B^[5]^. The Fe^3+^ is then transported into the enterocytes through divalent metal transporter 1 (DMT1), also known as SLC11A2^[6]^ (Figure 1A). Beyond the GI epithelium, DMT1 is ubiquitously expressed and plays an essential role in cellular iron uptake (discussed later in Section 2.1.2). Once inside the cell, iron can either be bound to chaperone proteins for subcellular trafficking and storage through poly(rC)-binding protein (iron chaperone) and ferritin, respectively, or exported out of the cells and into the systemic circulation^[7]^. Colorectal cancer (CRC) cells overexpress DMT1 and disruption of DMT1 significantly reduces tumor number and burden^[8]^.

Heme from dietary sources or hemolysis represents another major iron source, although the molecular mechanisms of intestinal heme absorption remain incompletely defined. HRG1 (SLC48A1) is a validated heme importer in macrophages and is expressed in duodenal enterocytes, making it a strong candidate for dietary heme uptake^[9]^. More recently, a CRISPR activation screen identified SLCO2B1 as a heme transporter in certain contexts, though its expression in intestinal tissue is limited^[10]^ (Figure 1A). Heme-binding proteins such as haptoglobin and hemopexin scavenge hemoglobin- and heme-containing complexes in the bloodstream. These complexes are internalized through receptors including CD163 and LRP1/CD91, which are upregulated in several tumor types, enabling them to tap into systemic heme reserves^[11,12]^. The role of heme in cancer likely extends beyond iron supply. Heme degradation via heme oxygenases (HOs) (HO-1/HO-2) yields biliverdin, bilirubin, and carbon monoxide, molecules with antioxidant and signaling properties that may buffer iron-driven oxidative stress and promote tumor survival^[13]^. Whether increased heme availability primarily contributes iron or directly alters signaling pathways remains an important open question.

#### 2.1.2 Cellular iron uptake

In the circulation, ferric iron is carried by transferrin (TF). Cells internalize TF-bound iron di-ferric complex via the transferrin receptor (TFRC/CD71), and endosomal acidification triggers iron release. Endosomal ferric iron is reduced to Fe^2+^ by STEAP family metalloreductases and transported into the cytosol through DMT1 expressed on the endosome membrane (Figure 1A). TFRC and apo-transferrin are then recycled back to the plasma membrane^[7]^. TFRC is among the most consistently upregulated iron-handling proteins in cancer and is associated with poor prognosis in many malignancies^[14]^. Its high expression reflects both iron demand and proliferative signaling. Disruptions in endosomal acidification or trafficking, including inhibition of V-ATPases or endosome–lysosome fusion, impair processing TF-bound iron, induce intracellular iron deficiency, and are being investigated as a potential therapeutic strategy^[15]^.

In addition to the transferrin receptor, cancer cells import iron through lipocalin-2 (LCN2) and its receptor LCN2R/SLC22A17, which bind siderophore-iron complexes. LCN2 is often elevated in inflammatory tumors and contributes to iron acquisition within the tumor microenvironment^[4]^. A transmembrane glycoprotein CD44, identified as a cancer stem cell marker, can facilitate uptake of iron containing hyaluronates during the epithelial mesenchymal transition independent of TFRC expression^[16,17]^ (Figure 1A).

Ferritin, an iron storage protein, is secreted into the blood and therefore can serve as an alternative source of iron. While the source of serum ferritin remains unclear, surface receptors such as SCARA5 and TIM2 have been shown to facilitate endocytosis of iron bound serum ferritin and deliver iron to the cytosol through the endosomal pathway described above^[18,19]^. Cancer cells can effectively take up ferritin which is secreted into the tumor microenvironment by tumor associated macrophages and other non-cancer cells.

### 2.2. Iron trafficking

Once iron enters the cytosol, it can be directed to specific organelles, sequestered by iron-binding chaperones, or incorporated into the metabolically active LIP. The distribution of iron among these fates is coordinated by intracellular trafficking pathways that match iron availability with metabolic demand (Figure 1A). Perturbations in these pathways are highly detrimental: expansion of the LIP promotes Fenton chemistry and oxidative injury, whereas contraction of the LIP leads to functional iron deprivation and impaired enzymatic activity^[20]^.

Cancer cells with an expanded LIP are highly susceptible to iron-driven oxidative stress and rely on multiple redox-buffering systems to limit ROS accumulation. Thus, defining the mechanisms that regulate intracellular iron trafficking and understanding how these processes are rewired in cancer may reveal vulnerabilities that can be exploited for therapy.

#### 2.2.1 Inter-organelle transport

Most iron uptake mechanisms deliver iron to the cytosol, where it is subsequently redistributed to subcellular compartments and organelles, including mitochondria, endoplasmic reticulum (ER), and the nucleus, to support metabolism, protein and lipid biosynthesis, and DNA replication^[1]^. Mitochondria also serve as the primary site for incorporation of iron into iron-sulfur (Fe-S) clusters and heme, essential iron-containing cofactors that are subsequently redistributed throughout the cell to client apo-proteins across various organelles^[1,21]^. This makes mitochondria a central hub for intracellular iron trafficking, utilization, and cofactor export. Given the higher metabolic turnover, cancer cells frequently exhibit elevated mitochondrial iron levels compared with normal cells^[22]^. Similar to the cytosolic LIP, excess mitochondrial iron can also generate ROS and trigger cell death.

Fe-S cluster and heme biosynthesis occur in the mitochondrial matrix, requiring iron transport across both the outer and inner mitochondrial membranes (OMM and IMM). While DMT1 is proposed to mediate iron transport across the OMM, mitoferrins 1 and 2 (MFRN1/SLC25A37 and MFRN2/SLC25A28) facilitate transport across the IMM^[23,24]^ (Figure 1B). Although these isoforms exhibit some functional redundancy, they display distinct tissue- and cell-specific expression profiles. Since the majority of Fe-S clusters and heme containing proteins are involved in mitochondrial respiration, defects in these biosynthetic pathways induce energy deficits, increasing mitochondrial iron by triggering an iron starvation response (discussed later in Section 2.4.1)^[1]^.

Beyond Fe-S and heme-binding proteins, approximately 34% of iron-binding proteins complex directly with inorganic iron ions as mono- or dinuclear centers, independent of mitochondrial involvement. Nonetheless, protein chaperones such as PCBP1 play a key role in mediating iron incorporation into apo-proteins, thereby limiting iron reactivity^[7]^. PCBP1 can coordinate three Fe(II) ions stabilized by glutathione (GSH) and selectively interact with target apo-proteins to facilitate proper metallation^[25]^.

#### 2.2.2 Iron storage

Due to its highly reactive chemical nature, iron must be complexed with proteins when not actively engaged in biological processes to minimize the LIP and prevent oxidative damage. Within cells, iron is primarily stored in ferritin nanocages, which are composed of 24 polypeptide chains that self-assemble into shell-like structures capable of storing up to 4,000 ferric ions (Figure 1A). Predominantly cytosolic, ferritin is a heteromer consisting of ferritin heavy chain (FTH1) and ferritin light chain (FTL) subunits. FTH1 contains a di-iron center that oxidizes Fe(II) to Fe(III), while FTL provides structural support by forming nucleation sites for mineralization. The ratio of FTH1 to FTL subunits within the 24-mer ferritin complex varies by cell type and determines the rate of iron mineralization^[26]^. Ferritin levels are tightly regulated at both transcriptional and posttranscriptional levels in response to cellular iron availability, oxidative stress, and oncogenic signaling, as discussed later. Similarly, excess mitochondrial iron is stored in a homopolymer of mitochondrial-specific ferritin, mitochondrial ferritin (FTMT)^[27]^. Under normal physiological conditions, FTMT plays a minimal role; however, in cancer cells with elevated mitochondrial iron, FTMT is thought to be critical for mitigating mitochondrial ROS and preventing oxidative damage^[28]^.

### 2.3 Iron export

Proper regulation of intracellular iron requires a balance between import and export, as disruption of this balance can compromise cellular function. Multiple mechanisms mediate iron export, many of which are altered in cancer to favor iron retention and support increased metabolic demands.

Iron efflux in mammalian cells is primarily mediated by ferroportin (FPN, also called SLC40A1) (Figure 1A). FPN is expressed on the basolateral membrane of intestinal epithelial cells, reticuloendothelial macrophages, and hepatocytes, where it regulates systemic iron homeostasis through dietary iron absorption, iron recycling, and maintenance of circulating iron levels, respectively. FPN expression is tightly controlled at both transcriptional and post-transcriptional levels by cellular and systemic iron availability, oxidative stress, and inflammatory cues^[29]^. Since FPN levels respond directly to intracellular iron, this downregulation indicates that upstream iron-sensing pathways are disrupted in cancer, promoting iron retention. In multiple cancers, including colorectal, breast, prostate, ovarian, and lung cancers, FPN expression is significantly downregulated, a change associated with poor prognosis and reduced survival^[4]^. In CRC, for example, cells aberrantly express the hepatic hormone hepcidin, which triggers internalization and degradation of FPN, further limiting iron export^[30]^.

Effective ferroportin-mediated export also requires multi-copper ferroxidases such as hephaestin (HEPH) or ceruloplasmin (CP), which oxidize Fe(II) to Fe(III)^[29]^. Oncogenic signals, including overexpression of the histone methyltransferase G9a, can suppress HEPH expression, further reducing iron efflux^[31]^.

Beyond FPN, iron can also be exported through ferroportin-independent pathways, primarily involving secretion of iron-loaded ferritin. This occurs via secretory autophagy or endosomal micro-autophagy, processes that deliver ferritin to the extracellular space involving CD63^[32]^. In breast cancer cells, ferroptosis inducers trigger formation of ferritin-containing endosomes via the membrane glycoprotein PROM2, effectively reducing intracellular iron and protecting cells from iron-mediated toxicity^[33]^. Extracellular ferritin can act as an iron carrier to neighboring cells and, in healthy liver tissue, is cleared by Kupffer cells. However, in metabolic disorders such as MASLD and hepatic steatosis, where Kupffer cell numbers are reduced, extracellular ferritin is instead taken up by ROS-sensitive hepatic stellate cells, promoting inflammation, fibrosis, and potentially hepatocellular carcinoma^[34]^. Despite its relevance, the mechanisms and regulation of endosomal ferritin export in cancer remain largely unexplored.

Iron can also exit the cell in the form of heme. Heme transporters, such as FLVCR1 in humans, mediate heme export, often in an adenosine triphosphate (ATP)-dependent manner^[35]^ (Figure 1A). While heme transport has been well characterized in model organisms like worms, the regulation and functional significance of heme export in cancer cells remain poorly understood, representing another important avenue for future investigation^[36]^.

### 2.4 Iron utilization

Iron is abundant in cells, and due to its unique redox properties, participates in numerous reactions essential for critical cellular processes. Iron-containing cofactors such as Fe-S clusters and heme, synthesized predominantly in the mitochondria, are coordinated by cysteine or histidine residues in apoproteins distributed throughout the cell^[1]^ (Figure 1B). In cancer cells, altered metabolic demands drive changes in iron-containing protein expression not only in mitochondria but across all cellular compartments.

#### 2.4.1 Mitochondrial function

Mitochondrial outer membrane Fe-S proteins, such as CISD1 and CISD2, regulate mitochondrial iron homeostasis and are upregulated in breast cancer cells^[37]^. Other proteins, including PINK1 and PRKN, which are key mitophagy regulators, reduce expression of mitochondrial iron importers SLC25A37 and SLC25A28, consequently suppressing KRAS-driven pancreatic tumor growth^[38]^. Conversely, inhibition of PINK1 or PRKN leads to mitochondrial iron overload and promotes tumorigenesis in pancreatic cancer models, an effect reversible by iron chelators, highlighting the critical role of mitochondrial iron levels^[39]^.

##### 2.4.1.1 Fe-S biosynthesis

Fe-S clusters are essential cofactors for ~21% of all cellular iron-binding proteins, participating in catalytic reactions, electron transport, regulation, and structural stabilization of other proteins. Fe-S biogenesis occurs predominantly in mitochondria via a multi-step assembly pathway producing [2Fe-2S], [3Fe-4S], and [4Fe-4S] clusters (Figure 1B). Sulfur is extracted from cysteine by NFS1 in complex with FXN, ISD11, and ACP proteins. Nascent clusters are assembled on the scaffold protein ISCU and trafficked to client apoproteins in the ER, cytosol, and nucleus via chaperones that are not yet fully characterized^[40]^.

Fe-S assembly and stability are also sensitive to cellular oxygen and ROS levels. High ROS destabilizes exposed Fe-S clusters, while growth defects induced by Fe-S dysfunction can be rescued by reducing ambient O_2_^[41]^. This places Fe-S clusters at a critical intersection of iron and ROS regulation, influencing iron-mediated cell death and ferroptosis. *In vivo*, tumor microenvironment oxygen levels of solid tumors vary from 2-8%, rising to ~14% in oxygen-rich metastases niches, such as the lung^[42]^. Fe-S clusters likely facilitate metabolic adaptation to changing oxygen levels, supporting invasion and metastasis. For example, high NFS1 expression in lung cancer cells promotes metastasis, whereas partial NFS1 deletion reduces metastasis without affecting primary tumor growth^[43]^. Fe-S deficiency also impairs tumor growth by limiting key Fe-S-containing proteins, such as DNA polymerase ε, which is indispensable for proliferation of triple-negative breast cancer cells due to their high genomic instability and altered cell cycle checkpoint responses^[44]^. Moreover, defects in the Fe-S biosynthesis can directly activate iron regulatory proteins (IRPs) dependent iron starvation response, which increases the LIP by upregulating iron uptake and limiting iron storage and export, sensitizing cells to oxidative cell death^[45]^.

##### 2.4.1.2 Heme biosynthesis

Heme is another critical iron-containing cofactor, present in ~47% of total cellular iron-binding proteins. Heme-binding proteins participate in catalytic functions (~50%), electron transport (complexes II-IV), oxygen transport (hemoglobin, myoglobin), and regulatory roles (e.g., catalase, peroxidases)^[1]^. While cells can import iron as heme, which is metabolized by HOs to release free iron, all mammalian cells express enzymes for de novo heme biosynthesis in mitochondria (Figure 1B). Heme synthesis begins in the mitochondrial matrix with condensation of glycine and succinyl-CoA by ALAS (the rate-limiting step). The product, 5-aminolevulinic acid, is transported to the cytosol for protoporphyrin ring assembly, then returns to the mitochondria for insertion of iron by an Fe-S cluster containing ferrochelatase. Heme is subsequently distributed to apoproteins via FLVCR1b, and unbound heme is chaperoned by proteins such as GAPDH, HBPs, FABP, PGRMC, and GSTs to prevent cytotoxicity. Heme can also be exported into systemic circulation via FLVCR1a, where it is bound by extracellular heme carriers^[13]^.

ALAS is an Fe-S cluster containing enzyme, which catalyzes the rate limiting step of heme biosynthesis and exists as two isoforms: ubiquitously expressed ALAS1 and erythroid-specific ALAS2^[46]^. ALAS1 is transcriptionally repressed by heme and iron through a negative feedback loop involving heme-dependent recruitment of EGR1 corepressors NAB1/2 to the 5′ UTR of ALAS1 mRNA^[47]^. Its promoter and enhancer regions also contain binding sites for nuclear receptors such as CAR, PPAR, PXR, and PGC1α, linking ALAS1 expression to nutrient sensing and oncogenic signaling pathways^[48–50]^. ALAS1 activity also depends on TCA cycle-derived succinyl-CoA; TCA perturbations affect succinyl-CoA levels and thus ALAS1 activity, influencing heme biosynthesis^[51]^. Fe-S cluster availability also indirectly modulates heme synthesis via effects on ALAS, SDH, and KGDH activity^[52,53]^.

In tumors with high energy demands, alterations in heme uptake, synthesis, and export are common^[54]^. Heme degradation by HO-1 (ubiquitous) and HO-2 (induced by hypoxia inducible factors, HIF1/2α, and nuclear factor erythroid 2-related factor 2, NRF2) releases iron and reduces free heme, although the protective compared to the cytotoxic consequences of this process remain context-dependent. In some contexts, heme supports antioxidant responses via BACH1 and NRF2 signaling^[55]^. Overexpression of FLVCR1a facilitates mitochondrial heme export, preventing cytotoxic accumulation and maintaining ALAS1 activity^[56]^.

#### 2.4.2 Cell proliferation

Iron supports cell cycle progression and proliferation through several mechanisms, and enhanced iron uptake, redistribution, and retention is a hallmark of highly proliferative cells, including cancer cells^[2]^. Non-heme iron-binding proteins, such as α-ketoglutarate-dependent dioxygenases, catalyze Fe- and α-ketoglutarate-dependent hydroxylation of proteins or nucleotides, mediating post-translational and epigenetic modifications in response to iron availability, respectively^[57,58]^. Most studied α-ketoglutarate-dependent dioxygenases include Prolyl Hydroxylases Domain enzymes and Tet methylcystosine dioxygenases (TET), involved in hypoxia signaling and epigenetic regulation. Accumulation of metabolites such as succinate and fumarate or other transition metals (e.g., nickel, cobalt) can inhibit these enzymes, promoting oncogenic signaling^[59,60]^. For example, in renal cell carcinoma, succinate/fumarate accumulation suppresses SDH and FH, that drives epithelial-mesenchymal transition. While TET enzymes regulate DNA and histone methylation, TET2 loss in acute myeloid leukemia (AML) derepresses heterochromatic regions and drives inflammation, reversible by ascorbic acid supplementation^[61–63]^. Iron-dependent histone demethylases, such as Jumonji domain-containing protein 2 C (JMJD2C) and Jumonji domain-containing protein 3, regulate oncogenic transformation in breast cancer and T-ALL, respectively^[64,65]^.

Iron is also a cofactor for ribonucleotide reductase, which catalyzes the conversion of ribonucleotides to deoxyribonucleotides, directly supporting DNA replication^[66]^. Iron chelation impairs S-phase progression, likely through inhibition of ribonucleotide reductase or Fe-S-containing DNA polymerases^[44]^. Intracellular iron levels, sensed by IRP1/2, modulate cell cycle regulators such as p21 and p27, leading to G_0_/G_1_ arrest, though the exact mechanisms are unclear, and they may involve transcription factors such as Sp1, ERα, c-Jun, p53, and KLF6^[67,68]^.

#### 2.4.3 Genomic integrity

Iron is essential for maintaining high proliferation rates and DNA replication fidelity. Many DNA polymerases, helicases (XPD, FANCJ, DNA2, RTEL1), glycosylases, and primases require Fe-S clusters for proper function^[69]^. Cancer cells’ high replication stress creates a dependence on iron-driven DNA repair machinery. Pharmacological targeting of iron-dependent enzymes, such as ribonucleotide reductase inhibition by gemcitabine or clofarabine, reduces proliferation in ovarian, breast, lung, pancreatic cancers, and ALL^[70]^. Iron also regulates p53 activity. Heme-bound p53 is degraded, whereas iron responsive element binding protein 2 (IRP2)-mediated regulation under iron deficiency can stabilize p53 transcripts^[71,72]^. Thus, the effect of iron on genomic integrity depends on its form and context.

## 3. Systemic Regulation

**Figure 2.**
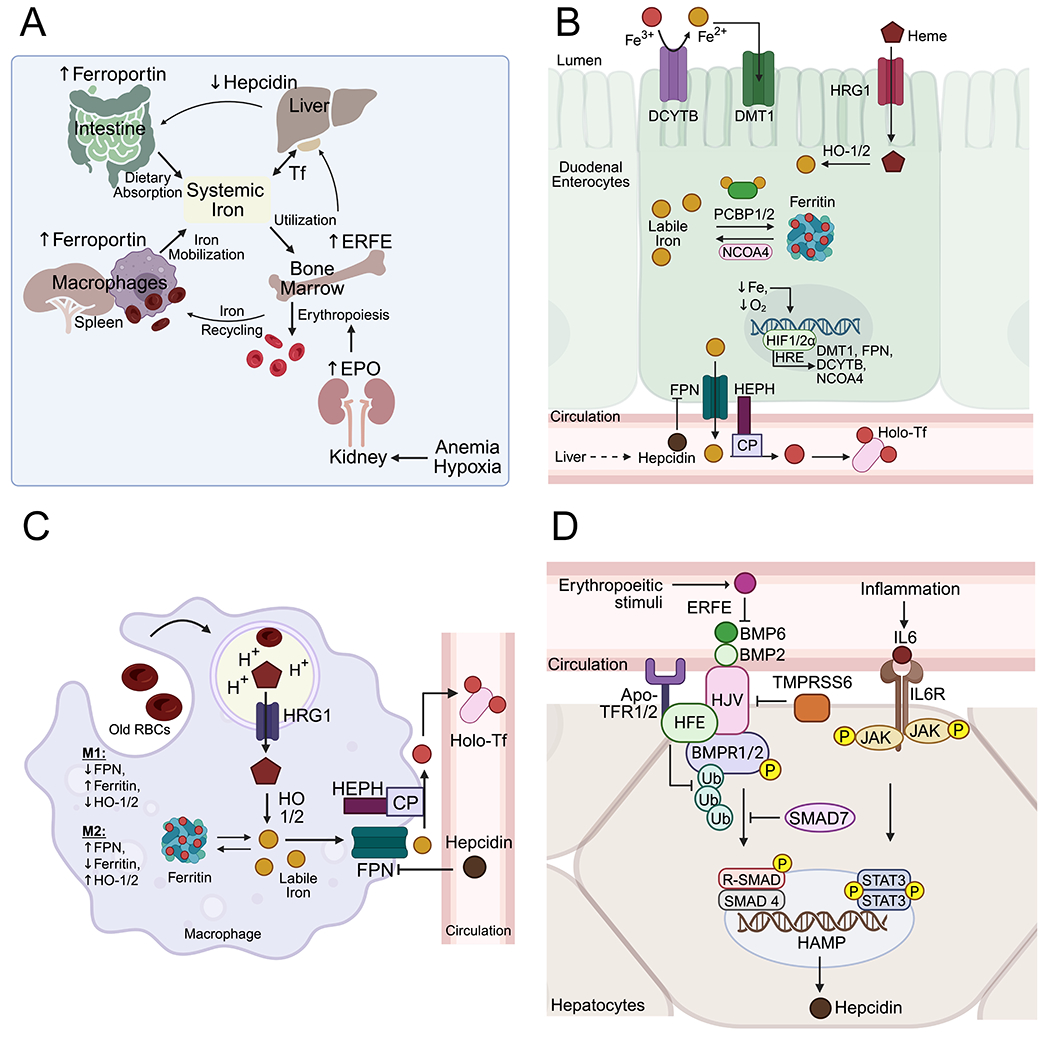
Systemic iron homeostasis. (A) Cross talk between intestine, liver, bone marrow, kidneys and spleen to regulate systemic iron homeostasis; (B) Regulation of intestinal iron absorption; (C) Iron recycling through reticuloendothelial macrophages; (D) Heaptic regulation of systemic iron homeostasis through regulated hepcidin expression. Created in BioRender.com. BMP2: bone morphogenetic protein 2; BMP6: bone morphogenetic protein 6; BMPR1/2: bone morphogenetic protein receptor type 1/2; CP: ceruloplasmin; DCYTB: duodenal cytochrome B; DMT1: divalent metal transporter 1 (SLC11A2); EPO: erythropoietin; ERFE: erythroferrone; HAMP: hepcidin antimicrobial peptide; HEPH: hephaestin; HFE: hemochromatosis protein; HIF2a: hypoxia-inducible factor 2 alpha; HJV: hemojuvelin; HO1/2: heme oxygenase 1/2; HRE: hypoxia response element; HRG1: heme responsive gene 1; IL6R: interleukin 6 receptor; JAK: Janus kinase; NCOA4: nuclear receptor coactivator 4; PCBP1/2: poly(rC) binding protein 1/2; R-SMAD: receptor-regulated SMAD (SMAD1/5/8); SMAD4: SMAD family member 4; SMAD7: SMAD family member 7; STAT3: signal transducer and activator of transcription; TFRC1/2: transferrin receptor 1/2; TMPRSS6: transmembrane serine protease 6 (Matriptase-2); Ub: ubiquitin.

Systemic iron levels are tightly regulated through crosstalk between distantly related tissues, coordinating iron absorption, utilization, recycling, and tissue distribution. The cellular iron regulatory mechanisms described above underlie this multi-organ coordination and exhibit tissue-specific differential regulation (Figure 2A).

### 3.1 Intestinal iron absorption

Regulation of dietary intestinal absorption is maintained by modulating the iron flux through intestinal enterocytes via regulation of apical and basolateral iron transporters in response to systemic iron levels and physiological demand^[73]^. Hepatocytes secrete hepcidin (HAMP), which suppresses iron uptake by inducing ferroportin internalization and degradation. FPN loss traps iron in enterocytes, leading to suppression of apical iron transporters, including DMT1, via inactivation of HIF2α and IRPs^[73,74]^.

During iron deficiency, anemia, or increased systemic demand, serum hepcidin levels drop, stabilizing ferroportin at the basolateral membrane and increasing iron flux^[29]^. Reduced cytosolic iron activates IRPs and HIFs, further upregulating DMT1, NCOA4, and FPN until systemic iron levels are restored^[7]^ (Figure 2B). FPN levels are significantly reduced in CRC cancer cells at the mRNA level and are further reduced through the autocrine action of hepcidin, discussed later^[30]^.

### 3.2 Hepatic regulation

The liver is central to systemic iron homeostasis, coordinating between the intestine, reticuloendothelial macrophages, kidneys, and bone marrow. Hepatocytes produce hepcidin, which reduces intestinal absorption and intracellular iron mobilization through macrophages by negatively regulating ferroportin^[75]^ (Figure 2C). Hepcidin binds ferroportin, inducing ubiquitination and proteasomal degradation^[76]^. Hepcidin deficiency results in iron overload and is sometimes treated with recombinant hepcidin, whereas excess hepcidin causes iron-refractory iron deficiency anemia, where oral iron supplementation is ineffective^[77]^. Hepcidin transcription is regulated by systemic iron levels, inflammation, and erythropoietic activity through the BMP-SMAD pathway. Dysregulation of this axis results in genetic iron overload syndromes^[78]^. Epidemiological studies link iron overload to increased risk of colorectal, lung, pancreatic, and breast cancers^[4]^. Hereditary hemochromatosis patients with high iron intake have a ~20-fold increased risk of hepatic and colonic malignancies^[79]^ (Figure 2D).

Hepcidin was initially identified as an acute-phase protein in septic patients, with IL-6-mediated JAK2-STAT3 activation driving its expression^[80]^. Therefore, chronic inflammation can also increase serum hepcidin levels, resulting in non-iron-deficiency anemia, as seen in cancer patients, characterized by low serum iron and low transferrin saturation^[81]^. In CRC, tumor cells can express hepcidin via HIF2α, acting in an autocrine fashion to suppress ferroportin-dependent export and retain intracellular iron to support iron-dependent metabolism^[30]^.

### 3.3 Renal regulation

Erythropoiesis is the largest systemic iron sink, with each erythrocyte containing ~3 million hemoglobin molecules (4 heme groups each). The erythroid compartment contains ~2/3 of total body iron and utilizes ~25 mg/day to maintain turnover^[82]^. Consequently, erythropoiesis is highly dependent on iron availability and strongly influences systemic iron homeostasis^[83]^. Renal erythropoietin (EPO) production is stimulated by low erythrocyte counts and tissue hypoxia via HIF2α activation^[84]^. EPO was initially thought to directly repress hepatic hepcidin, as their levels are inversely correlated. However, it is now established that EPO induces erythroferrone transcription in erythroblasts^[85]^. Erythroferrone suppresses hepatic BMP-SMAD signaling, downregulating HAMP transcription to facilitate iron absorption and mobilization in support of erythropoiesis^[86]^.

## 4. Cellular Regulation

Cellular iron levels are tightly maintained through coordinated regulation of proteins involved in iron import, storage, and export, responding dynamically to cellular iron demands and availability. Given iron’s central role in diverse metabolic processes and the potential deleterious effects of both deficiency and overload, multiple regulatory mechanisms ensure robust iron sensing. Alterations in these pathways are a hallmark of many cancer cells, enabling them to maintain high intracellular iron levels and exploit iron-rich environments to support proliferation and disease progression. Key regulatory mechanisms are discussed below.

### 4.1 IRP and iron response elements (IRE)

**Figure 3.**
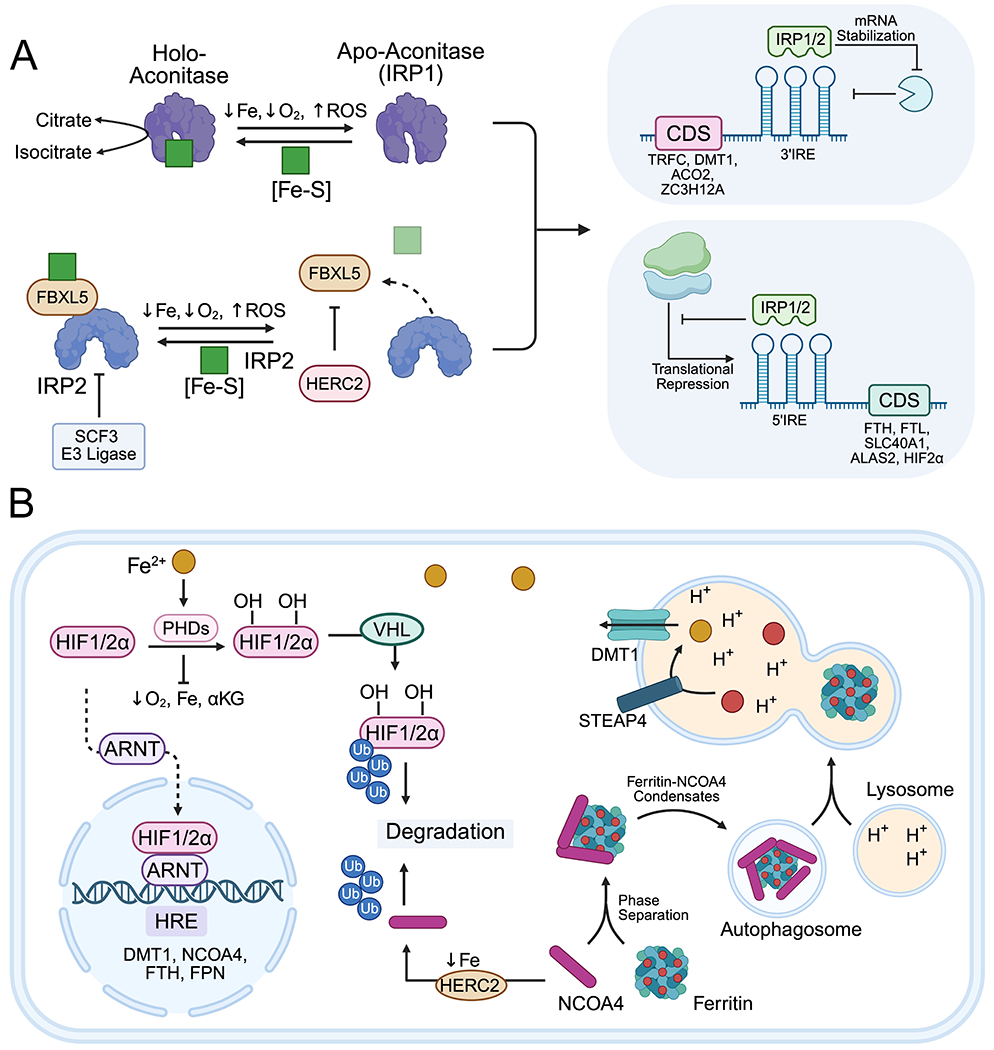
Regulation of cellular iron homeostasis. (A) IRE-IRP interaction; (B) Oxygen sensing and ferritinophagy. Created in BioRender.com. ACO2: aconitase 2; ALAS2: 5-aminolevulinate synthase 2; ARNT: aryl hydrocarbon receptor nuclear translocator; CUL3: cullin 3; DMT1: divalent metal transporter 1 (SLC11A2); FBXL5: F-box and leucine-rich repeat protein 5; FTH1: ferritin heavy chain 1; FTL: ferritin light chain; HERC2: HECT and RLD domain containing E3 ubiquitin protein ligase 2; HIF2a: hypoxia-inducible factor 2 alpha; HRE: hypoxia response element; IRE: iron-responsive element; IRP1/2: iron regulatory protein 1/2; NCOA4: nuclear receptor coactivator 4; PHD 1/2/3: prolyl hydroxylase domain 1/2/3; SCF3: skp, cullin, F-box containing complex 3; SLC40A1: solute carrier family 40 member 1 (ferroportin); sMAF: small musculoaponeurotic fibrosarcoma oncogene homolog; STEAP4: six-transmembrane epithelial antigen of prostate 4; TFRC: transferrin receptor; VHL: von hippel-lindau tumor suppressor.

Iron regulatory protein 1 (IRP1, also known as cytosolic Aconitase 1, encoded by ACO1) and IRP2, encoded by IREB2, constitute conserved iron-sensing circuits that post-transcriptionally regulate iron metabolism genes. Under low iron conditions, IRPs bind IREs, stem-loop structures located in the 5′ or 3′ untranslated regions of target mRNAs involved in iron import, storage, export, and utilization^[7]^ (Figure 3A).

#### 5′ UTR IREs:

Binding of IRPs to 5′ UTR IREs suppresses translation by blocking ribosomal subunit assembly. Genes with 5′ IREs typically encode iron storage, export, and utilization proteins such as FTH1, FTL, SLC40A1, ALAS2, HIF2α, as well as CD63 (ferritin secretion), limiting iron sequestration and export to increase the LIP during deficiency^[87]^.

#### 3′ UTR IREs:

IRP binding stabilizes mRNA by protecting it from endonucleases such as Regnase-1(also known as ZC3H12A), and Roquin-1, increasing translation^[88,89]^. Genes with 3′ IREs include iron importers TFRC1 and DMT1, and Profilin 2 (negative regulator of TFRC endocytosis)^[90]^. IREs in HIF2α and ALAS2 mRNAs regulate renal erythropoietin expression and erythroid heme biosynthesis^[91,92]^. Putative IREs are also found in transcripts of metabolic genes, including mitochondrial ACO2 and cell cycle regulators, positioning iron as a key regulator of metabolism beyond its catalytic functions^[93]^.

Although IRP1 and IRP2 share overlapping targets, their regulation is distinct. IRP1 contains a [4Fe-4S] cluster under iron-replete conditions, functioning as a cytosolic aconitase. Low iron or oxidative stress destabilizes this cluster, converting IRP1 into an RNA-binding protein that regulates IRE-containing transcripts^[94]^. IRP2 does not bind iron cofactors directly but is regulated via the iron- and oxygen-sensitive FBXL5 protein, part of the SCF E3 ubiquitin ligase complex^[95]^. Under high iron, FBXL5 recruits IRP2 for ubiquitination and proteasomal degradation. FBXL5 stability depends on a di-iron hemerythrin-like domain and a [2Fe-2S] cluster in its leucine-rich repeat domain^[96]^. Low iron destabilizes FBXL5, resulting in IRP2 accumulation^[97]^.

Deletion of both IRP1 and IRP2 is embryonic lethal, whereas single knockouts display tissue-specific phenotypes affecting erythropoiesis, neurological function, and systemic glucose homeostasis^[98]^. Aberrant IRP activation in cancers promotes iron accumulation even under iron-replete conditions^[45]^. IRP2 deletion reduces tumor growth in breast cancer by lowering LIP, while IRP2 overexpression promotes iron-dependent tumor proliferation^[99]^. FBXL5 ablation in the liver leads to iron overload, oxidative stress, inflammation, and tumorigenesis^[100]^. Cisplatin disrupts IRP2-mediated iron sensing, and combining iron chelation with cisplatin enhances therapeutic efficacy^[101]^. Fe-S biogenesis defects and mitochondrial dysfunction can also activate IRPs independent of iron or oxygen, sensitizing cells to oxidative stress^[43]^. Thus, IRP activation enhances proliferation but also increases dependence on antioxidant pathways, presenting a potential therapeutic target.

### 4.2 Ferritinophagy

Beyond its role as an iron storage protein, ferritin contributes to both iron mobilization and export. Its expression regulates the size of the LIP, and lysosomal ferritin degradation replenishes cytosolic iron. Ferritinophagy, a selective autophagic process, is mediated by the chaperone NCOA4, whose activity is controlled by cellular iron levels^[102,103]^. NCOA4 oligomerizes and binds FTH1, forming a complex recognized by TAX1BP1 and delivered for macro-autophagy or endosomal micro-autophagy^[104]^. Iron loading onto ferritin destabilizes the NCOA4-FTH1 interaction and promotes NCOA4 degradation via HERC2, reducing ferritinophagy and lowering LIP^[105]^. During iron deficiency, NCOA4 transcription is upregulated by iron-sensing pathways, including HIF signaling (Figure 3B). Inhibition of ferritinophagy *in vitro* protects cells from oxidative stress and ferroptosis by reducing LIP^[106]^. NCOA4-deficient mice exhibit impaired iron mobilization from macrophages and hepatocytes, low serum iron, and increased reliance on dietary iron; these mice are resistant to iron overload and hemochromatosis^[107]^. Upregulated NCOA4-mediated ferritinophagy supports rapid proliferation, as observed in pancreatic ductal adenocarcinomas, driving mitochondrial respiration and resistance to RAS-MAPK inhibitors^[108]^. Targeting NCOA4 improves efficacy of MEK inhibitors and other chemotherapies^[109]^. However, excessive ferritin degradation can sensitize cancer cells to ferroptosis and oxidative stress, complicating therapeutic approaches^[110]^.

### 4.3 Oxygen dependent regulation

Iron and oxygen coordinate numerous metabolic reactions. Systemically, iron in heme enables oxygen delivery via hemoglobin, coordinating tissue cross-talk among intestine, liver, kidney, and bone marrow for absorption, circulation, and utilization^[29]^. At the cellular level, oxygen regulates iron-dependent target genes via HIF1α and HIF2α (Figure 3B). Under low oxygen and iron, HIFs induce genes containing hypoxia-response elements involved in glycolysis, angiogenesis, mitochondrial respiration, and iron regulation (e.g., TFRC, HO-1, NCOA4, HAMP). HIF stability is controlled by prolyl hydroxylase domain enzymes, which hydroxylate HIFα subunits under iron- and oxygen-replete conditions, promoting VHL-mediated ubiquitination and proteasomal degradation. Iron or oxygen deficiency suppresses prolyl hydroxylase domain activity, enabling HIFα dimerization with HIFβ, nuclear localization, and target gene activation^[111]^. HIF2α is further regulated during iron starvation via IRP1/2 binding to its 5′ UTR IRE^[112]^. Chronic HIF activation in hypoxic tumors promotes iron uptake, retention, metabolic remodeling favoring glycolysis and PPP, vascularization, invasion, and metastasis^[113]^.

## 5. Mechanisms of Iron Toxicity

**Figure 4.**
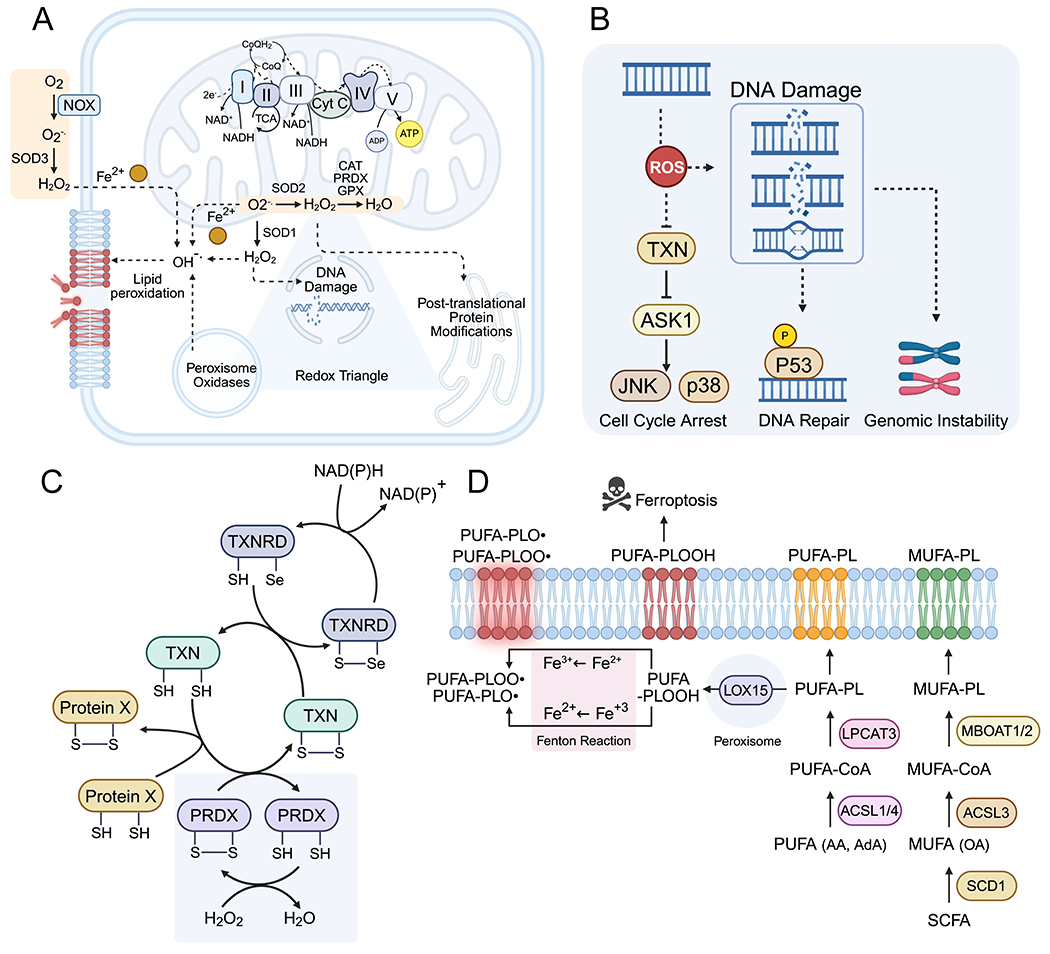
Mechanisms of iron induced oxidative stress. (A) Iron catalysed ROS generation and associated oxidative stress; (B) DNA oxidation; (C) Protein oxidation and peroxiredoxin relay system; (D) Lipid peroxidation. Created in BioRender.com. ACSL1/3/4: acyl-coA synthetase long chain family member 1/3/4; ASK1: apoptosis signal-regulating kinase 1; CAT: catalase; GPX: glutathione peroxidase; JNK: c-Jun N-terminal kinase; LOX15: 15-lipoxygenase; LPCAT3: lysophosphatidylcholine acyltransferase 3; MBOAT1/2: membrane bound O-acyltransferase domain containing 1/2; MUFA: monounsaturated fatty acid; MUFA-PL: monounsaturated fatty acid-phospholipid; NAD: nicotinamide adenine dinucleotide (oxidized form); NADH: nicotinamide adenine dinucleotide (reduced form); NOX: NADPH oxidase; p38: p38 mitogen-activated protein kinase; p53: tumor protein p53; PRDX: peroxiredoxin; PUFA: polyunsaturated fatty acid; PUFA-PL: polyunsaturated fatty acid-phospholipid; PUFA-PLO: polyunsaturated fatty acid-phospholipid olefinic (unsaturated); PUFA-PLOO: polyunsaturated fatty acid-phospholipid peroxyl; PUFA-PLOOH: polyunsaturated fatty acid-phospholipid hydroperoxide; ROS: reactive oxygen species; SCD1: stearoyl-coA desaturase 1; SOD 1/2/3: superoxide dismutase 1/2/3; TXN: thioredoxin; TXNRD: thioredoxin reductase.

Among transition metals, only iron and copper readily accept and donate electrons under physiological conditions. This redox flexibility is essential for normal cellular metabolism, but when uncontrolled, it generates ROS, contributing to tissue inflammation, cytotoxicity, and carcinogenesis (Figure 4).

ROS is a broad term describing molecules derived from molecular oxygen, including superoxide anion, hydrogen peroxide (H_2_O_2_), and hydroxyl radicals (·OH), and is part of a larger family of reactive species encompassing nitrogen, sulfur, carbon, selenium, and halogen derivatives^[114]^. These species act as signaling molecules, regulating stress responses, redox homeostasis, and adaptive cellular programs. However, accumulation of chelatable redox-active iron can drive ROS to supraphysiological levels, causing oxidative damage to DNA, lipids, and proteins and triggering ROS-dependent cell death^[115]^. Fe^2+^ can react with molecular oxygen to form superoxide anions (O_2_·^−^), which are subsequently converted to H_2_O_2_ by superoxide dismutase^[20]^. Moreover, spontaneously redox cycling of free labile iron between Fe^3+^ and Fe^2+^ in the presence of H_2_O_2_ generates highly reactive ·OH via the Fenton and Haber-Weiss reactions. The resulting ·OH are among the most reactive ROS in cells, capable of oxidizing lipids, proteins, and nucleic acids. Due to their extreme reactivity, conventional antioxidants are largely ineffective, and enzymatic removal of H_2_O_2_ by peroxidases such as catalase is a primary cellular defense^[114]^.

Mechanistically, iron induced cell death can be classified as either nonspecific oxidative cell death or ferroptosis, a form of regulated cell death driven through iron dependent lipid peroxidation. While conceptually there are significant overlaps between the two pathways, unlike non-specific oxidative cell death, sensitivity to ferroptosis is dependent on several intrinsic factors such as phospholipid composition, cellular metabolism, and antioxidant capacity^[114]^ (Figure 4A). Whether lipid peroxidation is driven predominantly by non-specific Fenton chemistry or by iron-dependent enzymatic reactions such as lipoxygenases remains an open question.

### 5.1 Non-specific oxidative cell death

Electron transfer through the electron transport chain converts the redox potential of NADH, NADPH, and FADH_2_ into a proton motive force across the inner mitochondrial membrane, ultimately generating ATP. While ~98% of oxygen is reduced to water efficiently, ~2% undergoes partial reduction, producing mitochondrial ROS^[116]^. Disruption of the electron transport chain function or accumulation of mitochondrial iron can exacerbate ROS generation, highlighting the necessity for tightly coordinated mitochondrial iron uptake, Fe–S and heme biosynthesis, and export processes^[117,118]^. Additional mitochondrial ROS sources include electrophilic TCA cycle intermediates such as fumarate and itaconate^[119]^.

Highly proliferative cells, including many cancer types, often rely on glycolysis for energy and anabolic precursors, which reduces mitochondrial ROS production but introduces reactive carbonyl byproducts like methylglyoxal. Emerging evidence challenges the classical Warburg effect, showing that some cancer cells simultaneously upregulate glycolysis and ETC components, increasing mitochondrial ROS^[120]^. In this revised model, glucose fuels glycolysis while OXPHOS is supported by amino acids (e.g., serine, glutamine) and fatty acid β-oxidation. These metabolic adaptations reshape the mitochondrial proteome and iron homeostasis, influencing susceptibility to oxidative cell death, including ferroptosis.

Mitochondrial ROS also function as signaling molecules, modulating responses to hypoxia, nutrient availability, and growth factors. Partial oxygen reduction under hypoxia, a hallmark of solid tumors, increases mitochondrial ROS, which can activate HIF1/2α independently of oxygen or iron levels^[121]^. This ROS-mediated HIF activation promotes angiogenesis via vascular endothelial growth factor (VEGF) expression and supports pro-survival signaling through MAPK and AKT pathways, suppressing apoptosis^[121,122]^. Overall, iron-mediated redox cycling, ROS generation, and mitochondrial metabolism are tightly interconnected. Dysregulation of these processes contributes to oxidative stress, DNA and protein damage, and lipid peroxidation, which underlie ferroptosis and other forms of iron-dependent cell death. The balance between ROS production and antioxidant defenses, influenced by iron availability, cellular metabolism, and organelle crosstalk, is therefore a critical determinant of cell fate.

#### 5.1.1 DNA oxidation

Iron and iron-catalyzed ROS possess carcinogenic potential by oxidizing nucleotides, leading to DNA modifications and mutations. For example, the elevated frequency of G to T transversions observed in lung tumors of smokers is thought to result from oxidative DNA damage induced by ROS generated from carcinogenic epoxides^[123]^. In addition, ROS-mediated oxidation of thiol groups on cysteine and methionine residues of critical DNA repair proteins can indirectly compromise DNA integrity and trigger cell cycle arrest^[124,125]^. Perinuclear mitochondria are considered a major source of nuclear ROS, and histone demethylation by LSD1 also produces H_2_O_2_ as a byproduct, contributing to the nuclear ROS pool^[126,127]^. Elevated H_2_O_2_ production by NOX4 in AML promotes ROS-induced double-strand breaks and chromosomal aberrations, thereby driving tumorigenesis^[128]^ (Figure 4B).

DNA lesions are typically recognized and repaired by substrate-specific repair pathways; while nucleotide excision repair addresses bulky adducts, ROS-induced lesions are primarily corrected through base excision repair^[129]^. A common oxidative DNA modification, 7,8-dihydro-8-oxo-2’-deoxyguanosine, is recognized and excised by the glycosylase OGG1 in eukaryotic cells^[130]^. Loss of OGG1 increases spontaneous mutation rates in proliferative cells and accelerates malignant transformation in the liver^[131]^. Interestingly, oxidative DNA and RNA lesions can also serve regulatory functions by activating pro-inflammatory signaling, DNA damage sensing pathways, and cell cycle and apoptosis regulators^[132–134]^. For instance, guanine oxidation within promoter regions, such as VEGF, can alter HIF1α binding and transcriptional regulation, as observed in OGG1-deficient mice^[135]^. Beyond transcriptional effects, OGG1 bound to 8-oxo-2’-deoxyguanosine can interact with small GTPases, including Ras, Rac1, and Rho, with high affinity, activating downstream ERK1/2 and Raf-1 signaling^[136–138]^. These oxidative base modifications are detectable in plasma, urine, and tissue biopsies and can serve as biomarkers of oxidative DNA damage. Furthermore, oxidative DNA damage and the subsequent repair processes generate strand breaks, DNA adducts, excision repair intermediates, and crosslinks, which can be assessed by single-cell electrophoresis and exploited for cancer diagnosis.

#### 5.1.2 Protein oxidation

Similar to DNA, proteins are susceptible to oxidation by cellular ROS, which can induce structural and conformational changes, alter enzymatic activity, modulate protein-protein interactions, or trigger protein degradation. Among ROS-mediated protein oxidations, H_2_O_2_ is the most extensively studied due to its relative stability and moderate reactivity. In mitochondria, H_2_O_2_ is generated under physiological conditions through the reaction of H_2_O with superoxide produced on the matrix side by Complexes I and III of the electron transport chain, including reverse electron transport^[121]^. Mitochondrial peroxiredoxins (PRDX), reduce H_2_O_2_ to water but can also release it into extramitochondrial compartments^[139]^. Although H_2_O_2_ release has been mainly studied in isolated mitochondria, its behavior as a cytosolic oxidant *in vivo* remains incompletely understood. Additional cellular sources of H_2_O_2_ include peroxisomal enzymes, which contribute to redox signaling and metabolic regulation^[140]^.

H_2_O_2_-mediated post-translational modifications, particularly oxidation of cysteine and methionine residues, regulate the redox metabolism of cancer cells. Due to the relatively low reactivity of H_2_O_2_ with thiols in the absence of catalysts, non-specific protein oxidation and aggregation are uncommon; instead, most effects are mediated through peroxiredoxin-dependent post-translational modifications. H_2_O_2_, via PRDXs, regulates metabolic pathways, kinase signaling cascades, transcription factors, and cell survival programs. PRDXs serve dual roles as H_2_O_2_ scavengers and as mediators of protein-specific H_2_O_2-_dependent oxidation through disulfide exchange reactions^[141]^. The specificity of cysteine oxidation is thought to involve redox relay systems, in which oxidation signals are transferred from one protein’s cysteine residues to another. For example, oxidation of the NRF2 negative regulator KEAP1 induces its degradation, activating NRF2-dependent transcriptional programs and enhancing the cellular antioxidant response (Figure 4C). In mammalian cells, PRDX1 and PRDX2 oxidize apoptosis regulator apoptosis signal-regulating kinase 1 (ASK1) and the transcription factor STAT3, while ER-resident PRDX4 oxidizes protein disulfide isomerase, promoting disulfide formation and maintaining protein folding and quality control^[142]^. In cancer cells, H_2_O_2_-mediated oxidation of the phosphatase and tensin homologue suppresses its activity, inactivating pro-apoptotic pathways and sustaining PI3K/AKT signaling^[143]^.

### 5.2 Ferroptosis

Ferroptosis is a form of regulated cell death characterized by iron-dependent phospholipid peroxidation, which destabilizes cellular membranes, induces osmolytic stress, and ultimately triggers cell death. Unlike other forms of cell death, ferroptosis is strictly iron-dependent: iron supplementation can promote it, while iron chelation suppresses it^[144]^. Although iron contributes to other cell death pathways such as apoptosis and necroptosis, it is essential for driving ferroptosis^[145]^. Dysregulation of cellular iron homeostasis, particularly elevated LIP, is a common feature across many cancers, making ferroptosis inducers selectively cytotoxic to malignant cells. However, cancer cells often upregulate antioxidant systems and ferroptosis suppressors, complicating therapeutic applications.

#### 5.2.1 Lipid peroxidation

The hallmark of ferroptosis is lipid peroxidation, which is initiated and propagated by ROS, particularly hydroxyl radicals. While free iron can catalyze nonspecific ROS production, the absolute requirement for iron in ferroptosis indicates that enzyme-catalyzed lipid peroxidation is the dominant mechanism^[146]^. Non-enzymatic, free radical-mediated oxidation generates diverse lipid peroxides depending on the availability of bis-allylic hydrogens in polyunsaturated fatty acids^[147]^. In contrast, enzymatic peroxidation, mediated by lipoxygenases such as LOX15, selectively targets arachidonoyl-PE and adrenoyl-PE, requiring prior biosynthesis by acyl-CoA synthetase long-chain family member 4 and lysophosphatidylcholine acyltransferase 3^[148,149]^. Pharmacological or genetic disruption of LOX15 or Acyl-CoA synthetase long-chain family member 4 reduces ferroptosis sensitivity, underscoring the role of regulated, enzyme-mediated lipid oxidation^[150,148]^. Nevertheless, LOX15-deficient cells can still undergo ferroptosis, suggesting that both enzymatic and non-enzymatic lipid peroxidation contribute cooperatively (Figure 4D). Lipid peroxyl radicals propagate chain reactions, generating hydroperoxides and secondary products such as reactive aldehydes, including 4-hydroxynonenal, which serve as biomarkers of ferroptotic lipid damage^[151]^.

#### 5.2.2 Cellular metabolism

Cellular metabolic programs profoundly influence ferroptosis sensitivity through regulation of both iron and lipid availability. Cancer cells often exhibit elevated LIP alongside reprogrammed lipid metabolism, using fatty acid oxidation to fuel mitochondrial respiration while shunting glucose primarily through glycolysis. Accumulation of lipid antioxidants can protect against ferroptosis; for instance, loss of squalene monooxygenase in anaplastic large cell lymphoma increases squalene levels, acting as a radical-trapping antioxidant^[152]^. Similarly, 7-dehydrocholesterol and its biosynthetic enzyme, 7-dehydrocholesterol reductase, confer protection in neuroblastoma, Burkitt lymphoma, and hepatocellular carcinoma^[153]^.

Targeting metabolic regulators of lipid composition can sensitize cancer cells to ferroptosis. In KRAS-mutant lung adenocarcinoma, inhibition of fatty acid synthase prevents remodeling of oxidized lipids, enhancing susceptibility^[154]^. Multi-omic analyses in glioblastoma identified CDKN2A as a key regulator of oxidized lipids, with deletion increasing lipid peroxidation and ferroptosis vulnerability^[155]^. Interestingly, CDKN2A is a cell cycle regulator and therefore a tumor suppressor, and patients with homozygous CDKN2A deletion show high grade metastasis, therapeutic resistance, and poor prognosis^[156]^. However, it is possible that these CDK2NA deficient tumor cells patients would benefit from ferroptosis based therapies due to enhanced ferroptotic sensitivity. Additionally, metabolic pathways that alter mitochondrial respiration and iron homeostasis modulate ferroptosis sensitivity, integrating mitochondrial ROS production, polyunsaturated fatty acid availability, and cellular iron flux.

#### 5.2.3 Regulators of ferroptosis

**Figure 5.**
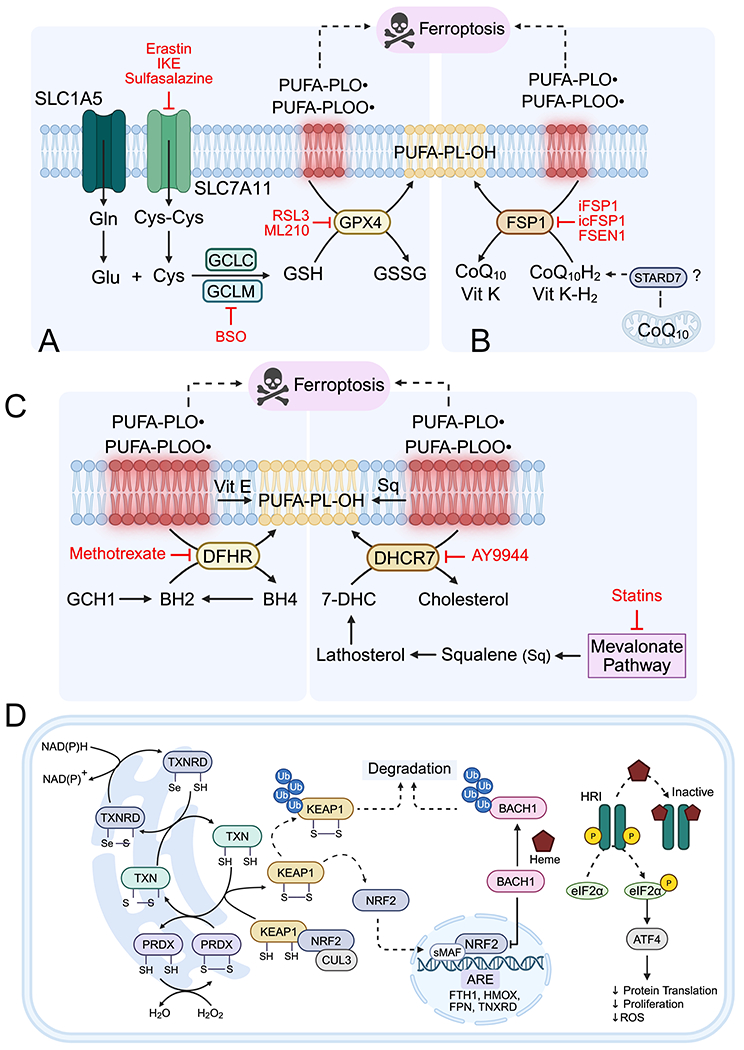
Cellular antioxidant defense systems. (A) SLC7A11-GPX4-GSH axis; (B) FSP1-NAD(P)H-CoQ axis; (C) GCH1-BH4 axis, cholesterol and mevalonate pathway; (D) NRF2 dependent redox signaling. Created in BioRender.com. 7DHC: 7-dehydrocholesterol; ATF4: activating transcription factor 4; BACH1: BTB and CNC homology 1; BH2: dihydrobiopterin; BH4: tetrahydrobiopterin; BSO: buthionine sulfoximine; CoQ10: coenzyme Q10 (ubiquinone-10); DFHR: dihydrofolate reductase; DHCR7: 7-dehydrocholesterol reductase; eIF2a: eukaryotic initiation factor 2 alpha; FSP1: ferroptosis suppressor protein 1; GCH1: GTP cyclohydrolase 1; GCLC: glutamate-cysteine ligase catalytic subunit; GCLM: glutamate-cysteine ligase modifier subunit; GPX4: glutathione peroxidase 4; GSH: glutathione (reduced); GSSG: glutathione disulfide (oxidized glutathione); HRI: heme-regulated inhibitor (EIF2AK1); KEAP1: Kelch-like ECH associated; NRF2: nuclear factor (erythroid-derived 2)-like 2 protein; PRDX: peroxiredoxin; PUFA-PL-OH: polyunsaturated fatty acid-phospholipid alcohol (hydroxyl); PUFA-PLO: polyunsaturated fatty acid-phospholipid olefinic (unsaturated); PUFA-PLOO: polyunsaturated fatty acid-phospholipid peroxyl; SLC1A5: solute carrier family 1 member 5 (glutamine transporter); SLC7A11: solute carrier family 7 member 11 (cystine/glutamate antiporter, xCT); STARD7: StAR-related lipid transfer domain containing 7; TXN: thioredoxin; TXNRD: thioredoxin reductase; ZC3H12A: zinc finger CCCH-type containing 12A.

Ferroptosis is an oxidative form of cell death and is induced by ROS generation. For aerobic animals, generation of ROS is a common consequence of running oxygen based mitochondrial respiration, and therefore cells have several antioxidant defense systems to protect against ferroptosis. Beyond antioxidants, regulation of cellular iron and lipid metabolism can also attenuate sensitivity to oxidative stress induced cell death (Figure 5).

##### 5.2.3.1 Antioxidants

Cellular antioxidants, including enzymatic and low-molecular-weight compounds, mitigate ferroptotic stress. Catalase converts H_2_O_2_ to water, while vitamin A, E, K, GSH, and cholesterol intermediates act as radical scavengers^[144]^. In vertebrates, the primary ferroptosis defense relies on the Xc^−^–GSH–GPX4 axis and the ferroptosis suppressor protein 1–CoQH_2_ system. GPX4, a GSH-dependent peroxidase, specifically reduces lipid hydroperoxides (PL-OOH) to alcohols, while Xc^−^ (SLC7A11) maintains intracellular cysteine for GSH synthesis. Depletion of GSH, cysteine, or inhibition of GPX4 sensitizes cells to ferroptosis, whereas iron chelation, cysteine supplementation, or synthetic radical-trapping antioxidants confer protection^[157]^. GPX4 activity depends on selenocysteine, which resists irreversible overoxidation, linking selenium metabolism and tRNA-mediated selenocysteine incorporation to ferroptosis regulation^[158]^. Additionally, GSH itself can bind Fe^2+^, acting as an iron carrier with antioxidant function independent of GPX4^[159]^ (Figure 5A).

FSP1 catalyzes reduction of CoQ10 to CoQH_2_ and vitamin K to hydroxyquinone, acting as lipophilic radical-trapping antioxidants^[160]^. Other mitochondrial enzymes, including dihydroorotate dehydrogenase, SQOR, and ETFDH, maintain CoQH_2_ pools, contributing to mitochondrial redox balance^[161–163]^ (Figure 5B). GTP cyclohydrolase 1 and tetrahydrobiopterin (BH4) constitute an additional, less-studied antioxidant axis, redistributing CoQ and conferring ferroptosis resistance, particularly in leukemias and lymphomas^[159]^ (Figure 5C). Cancer cells often display redundancy among these pathways, requiring combined targeting (e.g., GSH and thioredoxin inhibition) to effectively induce ferroptosis.

##### 5.2.3.2 Transcriptional and translational regulators

Ferroptosis is regulated by multiple antioxidant and metabolic programs controlled by redox-sensitive transcription factors. ROS-dependent regulation of the NRF2–KEAP1 axis is a central driver of the antioxidant transcriptional response, coordinating both cellular iron homeostasis and GSH synthesis via GCLC and GCLM^[164]^. Normally, NRF2 is continuously degraded by the E3 ligase adaptor KEAP1, a redox sensor. ROS oxidizes KEAP1 cysteines, releasing NRF2 to translocate to the nucleus and drive transcription of ~200 antioxidant response element genes, including FTH1, FTL, and SLC40A1, reducing LIP and oxidative damage^[165]^. KEAP1 loss-of-function mutations increase NRF2 activity, enhancing oxidative stress tolerance and correlating with poor prognosis in non-small cell lung carcinoma, papillary RCC, thyroid cancer, CRC, and ovarian cancers^[166]^ (Figure 5D). While NRF2-mediated suppression of ROS can prevent DNA damage and tumor initiation, in established tumors, it protects iron- and ROS-rich cancer cells from ferroptotic cell death^[165]^. A parallel antioxidant defense is mediated by BACH1, which is regulated by cellular heme rather than ROS, distinguishing it from NRF2 regulation^[55]^. Heme also activates the ATF4-dependent integrated stress response, inhibiting cap-dependent translation via HRI-mediated eIF2α phosphorylation and reprogramming transcription to mitigate stress, a process observed in hypoxic, nutrient-deprived cancer cells^[167]^ (Figure 5D).

Beyond canonical redox sensors, oncogenic signaling can modulate ferroptosis sensitivity by enhancing mitochondrial respiration and ROS production. MYCN amplification, observed in cancers such as neuroblastoma, increases intracellular iron flux, promoting lipid peroxidation and ferroptosis^[168]^. However, this elevated iron is not matched by sufficient antioxidant defenses, such as cysteine uptake, rendering MYCN-amplified neuroblastoma cells particularly sensitive to ferroptosis induced by cysteine deprivation. Similar to CDK2NA, MYCN also has a paradoxical effect on cell proliferation and tumorigenesis in glioblastomas. MYCN is an oncogene; its amplification is associated with poor survival outcomes and, therefore, ferroptosis based therapeutic intervention is a particularly attractive opportunity to treat otherwise highly aggressive tumors. Activation of the transcription factor YAP during epithelial-mesenchymal transition, triggered by loss of E-cadherin and Hippo pathway signaling, promotes ferroptosis. YAP upregulates intracellular iron and arachidonic acid, a substrate for lipid peroxidation, via increased TFRC and ACSL4 expression^[169]^. Ferroptosis sensitivity is further amplified during epithelial-mesenchymal transition and metastatic dissemination, as cells transition from the hypoxic tumor microenvironment into well-oxygenated tissues or systemic circulation, increasing ROS exposure^[170]^.

Nutrient-sensing pathways, particularly the mammalian target of rapamycin (mTOR), also modulate ferroptosis by regulating lipid composition. mTOR activation induces sterol regulatory element-binding proteins and stearoyl-CoA desaturase-1, promoting monounsaturated fatty acid biosynthesis^[171]^. Monounsaturated fatty acid lack bis-allylic hydrogens and are resistant to ROS-mediated oxidation, providing protection against lipid peroxidation. Conversely, activation of AMP-activated protein kinase, an mTOR inhibitor triggered by nutrient deprivation, reduces polyunsaturated fatty acid levels through suppression of acetyl-CoA carboxylase, protecting cells against ferroptosis^[172]^. Upstream activation of mTOR via the AKT pathway also confers protection by inhibiting GSK3β, which normally promotes NRF2 degradation^[170]^.

These observations highlight that ferroptosis sensitivity is context-dependent: the same signaling pathway can have opposing effects depending on upstream stimuli, the metabolic state, and the oxidative environment. This underscores the intricate interplay between metabolism, ROS, and cell death, emphasizing the need for further investigation into how redox and oncogenic signaling collectively modulate ferroptosis in cancer cells.

### 5.3 Other forms of regulated cell death

Iron, through its central role in regulating metabolism and ROS generation, can directly or indirectly influence other forms of regulated cell death, including apoptosis and necroptosis. Apoptosis and necroptosis are two major forms of regulated cell death triggered by intrinsic or extrinsic stimuli. With considerable crosstalk and overlapping mechanisms, apoptosis is characterized by cell shrinking, nuclear fragmentation, and membrane blebbing, while cells undergoing necroptosis swell up and die due to the breakdown of the plasma membrane. The intrinsic apoptotic pathway is activated in response to stimuli such as oxidative stress, DNA damage, and mitochondrial dysfunction. These cues trigger the release of cytochrome c from mitochondria, promoting apoptosome assembly and activation of executioner caspases^[173]^. Extrinsic apoptotic signals are mediated via engagement of surface receptors, such as tumor necrosis factor receptor 1 and FAS, by autocrine or paracrine soluble factors, including tumor necrosis factor alpha and FAS ligand^[20]^.

Iron-induced DNA damage, including single- and double-strand breaks and 8-oxoguanine lesions, activates the DNA damage response through ATM and ATR kinases, leading to p53 stabilization and ultimately apoptosis^[174]^. Beyond DNA damage, iron-dependent ROS can directly activate ASK1 via H_2_O_2-_mediated cysteine oxidation and inactivation of its negative regulators, including thioredoxin, glutaredoxin, and PRDX1^[175,176]^. Activated ASK1 initiates MAPK signaling, leading to activation of p38/JNK and pro-apoptotic BCL-2 family regulators^[177]^.

Intracellular iron also modulates extrinsic apoptotic pathways by influencing FAS splicing and surface expression. Low iron levels promote exon 6 skipping in the FAS transcript via activation of the iron-binding splicing factor SRSF7, reducing surface FAS expression and, consequently, FAS-dependent apoptosis^[178]^. Necroptosis, another regulated cell death pathway mediated through tumor necrosis factor receptor 1, FAS, and ROS-dependent JNK activation, is similarly influenced by iron-dependent regulation of FAS and ROS levels^[179]^. Notably, tumor necrosis factor alpha can increase the LIP and ROS by promoting ferritin degradation through JNK1 activation and enhancing NADPH oxidase 1 activity^[180]^. Under normal conditions, inflammatory NF-κB signaling induces FTH1 translation, suppresses ROS accumulation, and limits JNK activation^[181]^.

This convergence of ferroptosis, apoptosis, and necroptosis signaling pathways with iron metabolism underscores the multifaceted role of iron in regulating both cell survival and cell death.

## 6. Therapeutic Implications in Cancer

Cancer cells rely on iron-driven metabolism and tight regulation of redox homeostasis, creating vulnerabilities that can be therapeutically exploited^[182]^. Both modulation of iron availability and perturbation of antioxidant defenses can influence proliferation, disease progression, and sensitivity to oxidative cell death, including ferroptosis, apoptosis, and necroptosis^[183]^. Ferroptotic cancer cells show enhanced immunogenicity, while cytokines from the CD8^+^ T cells can further impair or induce critical ferroptotic regulators such as SLC7A11 and ACSL4 respectively, further driving ferroptosis in cancer cells^[184–186]^. However, excessive ferroptotic cell death can also deplete the highly sensitive immune cells including dendritic cells, CD8^+^ T cells, and macrophages, and therefore balancing the timing and dosing of ferroptotic agents in the context of immune check point inhibitors (ICIs) is critical to maximize the benefits and minimize non-specific toxicity^[187–189]^. Utilizing ferroptosis inducers in combination with existing treatment modalities beyond ICIs, such as radiotherapy and oncoprotein targeted molecules, has been shown to have a synergistic effect in various mouse models^[190,191]^. Over the last few years, several technological advances, such as new drug molecules and drug combinations, biomarker development, have improved understanding of the molecular mechanism of ferroptosis and oxidative cell death in the context of cancer. However, despite the success of ferroptosis inducers in preclinical models, translational success in the clinical remains limited due to pharmacological and biological limitations. The new emerging therapeutic strategies as well as challenges are discussed below.

### 6.1 Targeting iron availability

Manipulating intratumoral iron, either by depletion or supplementation, can slow cancer cell proliferation through suppression of iron-dependent metabolic pathways or induction of oxidative cell death, respectively. Traditional iron chelators, such as deferoxamine, are clinically effective in conditions such as thalassemia and hemochromatosis but show limited efficacy in hepatocellular carcinoma and leukemia^[192–194]^. More specific chelators, such as Dp44mT, a thiosemicarbazone derivative currently in clinical trials, bind iron while maintaining redox cycling and ROS generation, making them more potent at inducing tumor cell death^[195]^.

Gallium salts, including gallium maltolate and tris(8-quinolinolate)gallium(III), exploit iron-mimetic incorporation into iron-containing enzymes, inhibiting their catalytic activity and demonstrating anticancer properties^[196]^. However, cancer cells efficiently sequester intracellular iron from the extracellular environment, systemic circulation, and non-cancer cells in the tumor microenvironment via multiple regulatory mechanisms. For instance, CRC cells respond to dietary iron restriction or inhibition of intestinal absorption through DMT1 blockade by reducing tumor burden, but late-stage tumors may still acquire iron from increased vascularization^[8,14]^.

Conversely, inducing iron toxicity by increasing the LIP or suppressing antioxidant defenses can exploit cancer cells’ high baseline oxidative stress^[197]^. Iron supplementation in cells with elevated LIP can overwhelm antioxidant systems, leading to cell death. Iron oxide nanoparticles, such as Ferumoxytol (Feraheme), induce oxidative stress and reduce tumor burden in AML models^[198]^. Increased expression of iron transporters, such as TFRC1, can also be leveraged for targeted drug delivery, using transferrin-conjugated chemotherapeutics (e.g., doxorubicin, cisplatin) or toxins (e.g., diphtheria toxin, ricin) for cancer-specific cytotoxicity^[199]^.

### 6.2 Suppression of antioxidant signaling

The development of redox-based therapeutics aims to selectively exploit the dependence of cancer cells on antioxidant networks. A major challenge is modulating cancer-specific antioxidant activity while minimizing toxicity in normal tissues. Direct inhibition of redox-sensitive transcription factors (e.g., NRF2, BACH1, HIFs, MAPK, and ERK1/2) has shown limited clinical success due to their pleiotropic roles in the tumor microenvironment, angiogenesis, and immune modulation^[200–203]^. Targeting downstream effectors of these pathways offers a more precise approach.

GPX4 is a central ferroptosis suppressor, and its inhibition using electrophilic molecules such as RSL3, ML210, and ML162 can induce ferroptosis *in vitro*, though these compounds often exhibit off-target toxicity^[204]^. Novel approaches, including PROTAC-mediated GPX4 degradation, show improved *in vivo* efficacy^[205]^. GPX4 activity depends on GSH, and depletion of GSH via inhibition of SLC7A11 (e.g., erastin, sulfasalazine) or GCLC (e.g., buthionine sulfoximine) sensitizes cells to ferroptosis^[206,207]^. Enzymatic depletion of L-cyst(e)ine using engineered cyst(e)inase similarly induces ferroptosis in pancreatic cancer models^[208]^.

Other ferroptosis-suppressive pathways include FSP1-mediated reduction of CoQ10 to CoQH2 and BH4 synthesis via GTP cyclohydrolase 1. Inhibition of FSP1 (e.g., icFSP1) or BH4 synthesis (e.g., SPR inhibitors QM385, SPRi) enhances ferroptotic susceptibility. Functional redundancy among these pathways suggests that combinatorial targeting may provide broader therapeutic windows, depending on tumor type.

### 6.3 Oxidative metabolism

Mitochondrial metabolism is a key source of ROS, and its perturbation can selectively induce oxidative stress in cancer cells. Inhibition of dihydroorotate dehydrogenase, which reduces CoQ10 and supports nucleotide synthesis, induces replication stress and ferroptosis in cells with low GPX4 expression^[161]^. Disruption of selenium metabolism, such as targeting PRDX6, can indirectly impair GPX4 function by limiting selenocysteine availability^[209]^. The thioredoxin system, composed of thioredoxin and thioredoxin reductases, also regulates cellular and mitochondrial redox homeostasis and is often upregulated in cancers^[210]^. Inhibitors such as auranofin and Tri-1 suppress tumor growth in xenograft models, and their combination with GSH pathway inhibition synergistically induces cancer cell death^[211]^.

### 6.4 Limitations for clinical translation

In pathological conditions such as cancer, high iron demand and utilization shifts the balance between iron, iron catalyzed ROS, and antioxidant defense systems. In recent years, dysregulated iron and redox homeostasis has emerged as a hallmark of cancer, as cancer cells rely heavily on iron driven metabolism to sustain proliferation and disease progression. Inducing ferroptosis and oxidative cell death as a therapeutic strategy to selectively target cancer cells remains unachievable in the clinic due to several reasons.

#### Target specificity:

Inducing ferroptosis specifically in cancer cells while minimizing oxidative damage to highly sensitive normal tissues and immune cells in the TME continues to be the biggest hurdle in developing efficacious anti-cancer therapies. While inducing ferroptosis or iron toxicity in cancer cells can suppress tumor growth and progression, altering ferroptotic pathways may dampen the cytotoxic functions of tumor infiltrating immune cells^[212–214]^.

Cancer cells due to their increased intracellular iron and oxidative metabolism, are thought to be much more susceptible to ferroptosis inducers. However, this persistent oxidative stress leads to selective enrichment of resistant cancer cells with high antioxidant capacity in the TME^[215,216]^. Identifying and targeting these antioxidant defense mechanisms specific to cancer cells could induce synthetic vulnerability to ferroptosis inducers in cancer cells.

#### Redundant and compensatory defense mechanisms:

Chronic hypoxia and nutrient deficient TME imposes a selection pressure. Consequently, cancer cells develop a dependence on antioxidant defense systems, creating vulnerabilities that can be exploited for therapeutic interventions since limiting intra-tumoral iron availability is clinically challenging. However, the presence of several redundant and compensatory antioxidant signaling mechanisms makes this further complicating. Use of over simplified *in vitro* models used to identify the necessity of these pathways provides incomplete understanding of the physiologically relevant mechanisms. For example, inhibiting GPX4 or FSP1can lead to selection of cells overexpressing compensatory pathways or TME reprogramming, further driving the resistance against ferroptosis and oxidative cell death inducers^[217–219]^.

#### Intratumoral heterogeneity:

Several sub populations of cancer cells exist in the TME, which eventually disseminate from the primary tumor and lead to metastatic disease, the primary cause of cancer related morbidities. This intratumoral heterogeneity is crucial for cancer cells as it increases the chances of survival and positive selection in the TME^[220,221]^. However, this also poses a big challenge when developing therapies targeting cellular redox homeostasis. Different cells states vary vastly from a metabolic standpoint and therefore exhibit differential sensitivity to ferroptosis and oxidative cell death^[222–224]^. This is further confounded by pleiotropic roles of iron and iron catalyzed ROS, which can vary depending on the tissue of origin, heterocellular interactions within the tumor microenvironment, genetic and epigenetic heterogeneity.

#### Lack of defined biomarkers:

The success of a new drug relies on the design and execution of clinical trials which in turn depend on reliable biomarkers to identify the patient cohorts and monitor the disease progression. Ferroptosis based therapeutic strategies for cancer treatment however, remain limited due to an incomplete understanding of ferroptosis in a physiological context, as much of the current knowledge is derived from synthetic drug screens performed in *in vitro* models. Mechanistically, ferroptosis and oxidative cell death are characterized by the build up of short lived, highly unstable oxidants, which are extremely challenging to detect and quantify in real time. Most accurate real time detection methods rely on redox active fluorescent probes but require live cell imaging^[225,114]^. Over the last few years, use of mass spectrometry to detect stable products of these oxidation reactions in patient tissues, combined with a multi-omics approach (proteomics, lipidomics and metabolomics) to establish ferroptotic signatures has improved the understanding significantly^[226–230]^. However, given the complex and dynamic nature of redox biology and ferroptosis, translation of these physiological findings to the design of effective drug trials remains a challenge and therefore avoided by academic and industry.

## 7. Future Directions

Iron and iron containing cofactors (Fe-S clusters and heme) are crucial for cellular metabolism including cellular energetics, redox homeostasis and signaling, and cell proliferation. Iron metabolism is highly complex and is regulated at both the systemic and cellular levels to ensure adequate iron availability while preventing the toxicity associated with iron overload. While regulated production of ROS, catalyzed by labile iron, mediates several signaling processes, unchecked and unrestrained ROS production can be detrimental to cellular integrity. This regulation is achieved through coordination of iron uptake, storage, utilization, and export across different organs or organelles.

The studies summarized in this review highlight several decades of research that have significantly improved our understanding of iron homeostasis at the systemic and cellular scale.

Further research into the dynamic network of cellular and systemic iron regulatory pathways will strengthen our understanding of its profound implications for health and disease. A deeper understanding of the physiological role of ferroptosis and oxidative cell death is therefore critical for harnessing the therapeutic potential of ferroptosis or iron toxicity-based strategies while minimizing systemic toxicities. An interdisciplinary approach that integrates genetic and metabolic profiling on the cancer cells and the tumor microenvironment will be instrumental in identifying and exploiting inherent vulnerabilities to iron toxicity and ferroptosis in cancer.

## Data Availability

Not applicable.
